# Human brucellosis

**Published:** 2016

**Authors:** Viroj Wiwanitkit

**Affiliations:** 1 **Surin Rajabhat University, Babgkok, Thailand.**

**Keywords:** Brucellosis, Human, Zeoonosis

Dear Editor,

 The recent report on “human brucellosis” is very interesting (1). Indeed, brucellosis is a zoonosis. As noted by Roushan et al., this disease can be seen in “the Mediterranean areas, the south and the center of America, Africa, Asia, Arab peninsula, Indian subcontinent and the Middle East (1).” Mir et al. noted that this infection should be included in the list of differential diagnosis in case with fever of unknown origin (FUO) (2). The present great concern is the migration of the disease to the new setting. In Saudi Arabia, Aloufi et al. noted that there were reported cases among patients from nonendemic area (3). Bosilkovski et al. noted that the travelers risk in nonendemic areas would be considerable when visiting developing countries” where the endemic area is confirmed (4). In the author’s experience, in Southeast Asia, human brucellosis is a disease to be included in surveillance of possible new emerging disease. The first case was reported in 1970 (5) and the latest case in 2014 (6). Nevertheless, the previous local study on the seroprevalence of people living in close contact with goat still showed null prevalence (unpublished data, Rajadapisakesompote fund, Thailand, 2003). 

## Citation:

Wiwanitkit V. Human brucellosis. Caspian J Intern Med 2016; 7(2):232.
